# Preconception HbA_1c_ Levels in Adolescents and Young Adults and Adverse Birth Outcomes

**DOI:** 10.1001/jamanetworkopen.2024.35136

**Published:** 2024-09-24

**Authors:** Katharine J. McCarthy, Shelley H. Liu, Joseph Kennedy, Hiu Tai Chan, Frances Howell, Natalie Boychuk, Victoria L. Mayer, Luciana Vieira, Bahman Tabaei, Kacie Seil, Gretchen Van Wye, Teresa Janevic

**Affiliations:** 1 Department of Population Health Science and Policy, Icahn School of Medicine at Mount Sinai, New York, New York; 2 Department of Obstetrics, Gynecology, and Reproductive Science, Icahn School of Medicine at Mount Sinai, New York, New York; 3 Department of Health and Mental Hygiene, Bureau of Vital Statistics, New York, New York; 4 Department of Epidemiology, Mailman School of Public Health, Columbia University, New York, New York; 5 Division of General Internal Medicine, Department of Medicine, Icahn School of Medicine at Mount Sinai, New York, New York; 6 Department of Maternal and Fetal Medicine, Stamford Hospital, Stamford, Connecticut; 7 Department of Health and Mental Hygiene, Bureau of Health Equity, New York, New York

## Abstract

**Importance:**

Subclinical hyperglycemia before pregnancy may be associated with the likelihood of maternal morbidity but is understudied among young people.

**Objective:**

To explore the association of preconception hemoglobin A_1c _(HbA_1c_) levels among adolescents and young adults with risk of gestational diabetes at first live birth.

**Design, Setting, and Participants:**

This retrospective cohort study used linked 2009 to 2017 birth registry, hospital discharge, and New York City Department of Health A1C Registry data for birthing individuals aged 10 to 24 years with no history of diabetes and at least 1 preconception HbA_1c_ test in New York, New York. Statistical analysis was performed from August to November 2022.

**Exposure:**

Preconception HbA_1c_ values categorized as no diabetes (HbA_1c _<5.7%) or prediabetes (HbA_1c_ ≥5.7% to <6.5%).

**Main Outcomes and Measures:**

The primary outcome was gestational diabetes at first birth. Secondary outcomes included hypertensive disorders of pregnancy, preterm birth, cesarean delivery, and macrosomia. Log binomial regression was used to estimate the relative risk (RR) of gestational diabetes at first birth by preconception HbA_1c_ level, adjusting for prepregnancy characteristics. The optimal HbA_1c_ threshold for gestational diabetes was examined using receiver operating curve regression.

**Results:**

A total of 14 302 individuals (mean [SD] age, 22.10 [1.55] years) met study eligibility criteria. Of these, 5896 (41.0%) were Hispanic, 4149 (29.0%) were Black, 2583 (18.1%) were White, 1516 (10.6%) were Asian, and 185 (1.3%) had other or unknown race and ethnicity. Most (11 407 individuals [79.7%]) had normoglycemia before pregnancy, and 2895 individuals (20.2%) had prediabetes. Adjusting for prepregnancy characteristics, those with preconception prediabetes had more than twice the risk of gestational diabetes vs those with normoglycemia (adjusted RR [aRR], 2.21; 95% CI, 1.91-2.56). Preconception prediabetes was associated with small increases in the likelihood of a hypertensive disorder of pregnancy (aRR, 1.18; 95% CI, 1.03-1.35) and preterm delivery (aRR, 1.18; 95% CI, 1.02-1.37). The aRRs for cesarean delivery (aRR, 1.09; 95% CI, 0.99-1.20) and macrosomia (aRR, 1.13; 95% CI, 0.93-1.37) were increased but not statistically significant. The optimal HbA_1c_ threshold to identify gestational diabetes among adolescents and young adults was 5.6%. The threshold did not vary by obesity status but was slightly lower among Hispanic individuals (HbA_1c_ of 5.5%).

**Conclusions and Relevance:**

In this study of adolescents and young adults with at least 1 preconception HbA_1c_ test, prediabetes was associated with increased likelihood of maternal cardiometabolic morbidity at first birth. Efforts to optimize cardiometabolic health before pregnancy may avert excess maternal risk.

## Key Points

**Question:**

Is preconception hemoglobin A_1c_ (HbA_1c_) level associated with the likelihood of gestational diabetes and adverse birth outcomes among adolescents and young adults?

**Findings:**

In this cohort study of 14 302 New York City adolescents and young adults aged 10 to 24 years, preconception prediabetes (defined as HbA_1c_ ≥5.7% to <6.5%) was associated with more than twice the risk of gestational diabetes at first live birth and an 18% increased risk for both pregnancy hypertension and preterm delivery.

**Meaning:**

These findings suggest that adolescence and young adulthood may be a critical window to optimize cardiometabolic health before pregnancy and avert maternal and neonatal risk.

## Teaser

This cohort study evaluates the association of preconception hemoglobin A_1c_ (HbA_1c_) levels among adolescents and young adults with risk of gestational diabetes at first live birth.

## Introduction

Pediatric prediabetes, an intermediate stage of glucose intolerance before type 2 diabetes diagnosis, affects nearly 1 in 5 US adolescents aged 10 to 19 years and 1 in 4 young adults aged 20 to 24 years.^1,2^ A separate study^3^ examining trends among US youth found the prediabetes rate (fasting plasma glucose 100-125 mg/dL, oral glucose tolerance test 140-199 mg/dL, or hemoglobin A_1c_ [HbA_1c_] 5.7%-6.5%) doubled to affect nearly 1 in 3 adolescents aged 12 to 19 years between 1999 and 2018. In both age groups, Black and Hispanic individuals and those from low-income backgrounds bear the disproportionate burden.^3,4^ These trends are alarming, as adolescents and young adults with prediabetes generally exhibit a more adverse cardiometabolic risk profile, including elevated low-density lipoprotein cholesterol, elevated systolic blood pressure, central adiposity, and lower insulin sensitivity, than those with normoglycemia.^5^ The presence of these traits increases the risk of later life type 2 diabetes and cardiovascular disease in adult cohorts.^6,7^ Owing to the long follow-up period necessary, the association between adolescent prediabetes status and later life cardiometabolic risk, however, remains understudied.^8^

An earlier life phase that can signal a warning for latent or underlying cardiometabolic dysfunction is pregnancy.^9,10^ Gestational diabetes is transient glucose intolerance that is first recognized or emerges in pregnancy.^11^ Although gestational diabetes often resolves post partum, it confers a more than 10 times increased risk of type 2 diabetes in the next decade, with linear increases in absolute risk over time.^12,13,14^ Rates of gestational diabetes among young people have increased in recent years, from 16.0 to 22.4 cases per 1000 live births among adolescents aged 15 to 19 years and 29.9 to 40.7 cases among those aged 20 to 24 years between 2011 and 2019.^15^ These statistics suggest an escalation of the diabetes epidemic in later adulthood. The association between adolescent and young adult preconception prediabetes and the likelihood of gestational diabetes at first birth, however, is less known. Despite declines, the pregnancy rate among young people in the US still far exceeds that of other high-income countries, with 33 pregnancies per 1000 individuals aged 15 to 19 years and 112 pregnancies per 1000 individuals aged 20 to 24 years, and increasingly occurs among those who are low income.^16^ Given that the majority of pregnancies among adolescent (>75%) and young adults (>60%) are unwanted or mistimed^16,17^ and considering alarming cardiometabolic health trends in these age groups, greater clarity of the clinical significance of preconception prediabetes on pregnancy health is warranted. Yet, clinical guidelines for prediabetes care among pregnant individuals without other diabetes risk factors, including body mass index (BMI) greater than or equal to the 85th percentile, family history of type 2 diabetes, or minoritized race and ethnicity (ie, American Indian, Black, Hispanic, and Asian American or Pacific Islander) remain unclear.^1,18,19^ As a result, there are no consistent guidelines on whether and when to intervene following identification of prediabetes among those most at risk of unplanned pregnancy and, therefore, least likely to benefit from preconception counseling.

The lack of uniform treatment guidelines among adolescents and young adults with preconception prediabetes is, in part, attributable to little or mixed evidence on the importance of glucose measures during these age periods. The onset of puberty initiates dynamic cardiometabolic changes, including transient insulin resistance.^20,21^ Some cohorts of children and adolescents (<20 years old) with obesity have demonstrated lower discrimination ability of HbA_1c_ screening test, which is a nonfasting blood test that provides information about a person’s average blood glucose over the past 3 months compared with an oral glucose tolerance test, or fasting plasma glucose in identifying prediabetes and diabetes.^22,23,24^ Other evidence has demonstrated that 2-hour oral glucose and HbA_1c_ testing are equally valid in identifying dysglycemia in adolescents aged 10 to 18 years with obesity.^25^ However, HbA_1c_ validation studies conducted in pediatric populations to date have used HbA_1c_ thresholds extrapolated from adults and have largely not included a clinical comparison.^1,2^ Furthermore, little research has examined results specific to young adulthood (aged 20-24 years). This age period has been both considered part of adolescence^26,27^ and included in adult guidelines. The optimal threshold for HbA_1c_ prediabetes to identify the association with cardiometabolic-related complications in pregnancy in both adolescence and young adulthood has yet to be well characterized.

To address these gaps, we assessed how preconception HbA_1c_ risk levels among individuals aged 10 to 24 years inform the likelihood of gestational diabetes and related adverse maternal and neonatal outcomes at first birth. As a secondary objective, we tested the ideal categorization of preconception HbA_1c_ thresholds to project gestational diabetes at first birth, including whether the optimal threshold varied by traditional risk factors such as prepregnancy BMI or race and ethnicity as well as by age cohort.

## Methods

### Data Source

We obtained maternal data on individuals at first live birth between 2009 and 2017 from a retrospective population-based cohort study in New York City (NYC), the APPLE (A1c in Pregnancy and Postpartum Linkage for Equity) Cohort.^14,28^ This retrospective cohort was created by linking maternal data collected in several public surveillance data sets: (1) birth registration records, (2) hospital diagnoses (*International Classification of Diseases, Ninth Revision [ICD-9] *and *Tenth Revision [ICD-10]*) codes obtained from the Statewide Planning and Research Cooperative System (SPARCS), and (3) hemoglobin HbA_1c_ values and test dates from the HbA_1c_ Registry. Because this was a secondary analysis of existing data, informed consent was not obtained, in accordance with 45 CFR §46. This study was approved by the institutional review boards at the Icahn School of Medicine at Mount Sinai, the NYC Department of Health and Mental Hygiene, and the New York State Department of Health. The reporting of this manuscript adheres to the Strengthening the Reporting of Observational Studies in Epidemiology (STROBE) reporting guidelines.^29^

### Sample

The study sample included participants aged 10 to 24 years^26^ who had at least 1 HbA_1c_ test before conception (calculated using gestational age), no previous live birth, and no prior record of diabetes (via birth record, *ICD-9* 250.x and 648.0, or *ICD-10* E08-E13x, O24.0, O24.1, O24.3, O24.8, Z79.4 codes) or elevated HbA_1c_ test result (≥6.5%; to convert HbA_1c_ to proportion of total hemoglobin, multiply by 0.01) before pregnancy. We adopt the definition of 10 to 24 years to correspond with adolescent growth, which extends into early adulthood^26^; however, results disaggregated by 10- to 19-year-old and 20- to 24-year-old age cohorts are presented in the eTables 1 and 2 in Supplement 1. Birth records with missing data were excluded (169 individuals [1.2%]), as missing birth record data are unlikely to satisfy the missing at random assumption for multiple imputation.

### Exposure

Preconception HbA_1c_ thresholds were defined using existing classification criteria of (1) no diabetes (HbA_1c_ <5.7%) and (2) prediabetes (HbA_1c_ ≥5.7% to <6.5%).^2,30^ We also varied the classification of prediabetes using empirically informed cutoff points of HbA_1c_ 5.6% to less than 6.5% and HbA_1c_ 5.5% to less than 6.5% to assess the optimal categorization of subclinical HbA_1c_ levels among adolescents in relation to gestational diabetes.

### Outcomes

The main outcome was gestational diabetes (birth record indication, hospital record *ICD-9* 648.01-648.04 or *ICD-10* O24.4 codes) at first birth.^31^ We also examined other indicators of cardiometabolic dysfunction in pregnancy and associated adverse birth outcomes.^32^ These included hypertensive disorders of pregnancy (gestational hypertension *ICD-9* 642.3x, preeclampsia or eclampsia *ICD-9* 642.4x-642.6x and 642.7x via hospital diagnoses code, or birth record indication^33,34^) cesarean delivery via birth record, gestational age less than 37 weeks via birth record, or macrosomia^35^ (infant birth weight >4500 g via birth record).

### Covariates

Other maternal characteristics that were considered potential confounders between prediabetes and birth outcomes were adjusted for in the analytic models. These characteristics included age in years, race and ethnicity,^36^ whether the respondent had public or no insurance relative to private insurance, highest educational attainment (less than high school, high school degree or equivalent, some college, or college degree or higher), nativity (US born or born outside the US), smoking status the 3 months before pregnancy, and alcohol use during pregnancy (all variables ascertained via self-report on the birth record). We created the race and ethnicity variable by combining race and ethnicity information into the following categories: Asian, non-Hispanic Black (henceforth Black), Hispanic, and non-Hispanic White (henceforth White).^36^ We disaggregated Asian subgroups into South and Southeast Asian descent according to the mother’s reported country of origin,^37^ given evidence of higher gestational diabetes risk among South and Southeast Asian populations.^14,38,39^ Whether the respondent had public or no insurance relative to private insurance and whether the respondent had received any prenatal care in the first trimester were also reported via the birth record. We also adjusted for prepregnancy clinical characteristics and health behaviors that are potentially associated with preconception prediabetes and pregnancy complications, including prepregnancy BMI category in the birth record (calculated as weight in kilograms divided by height in meters squared; underweight, <18.5; normal weight, 18.5 to <25.0; overweight, 25.0 to <30.0; and obesity, ≥30.0) and preexisting chronic hypertension (*ICD-9* codes 401.x-405.x and 642.0x-642.2x or birth record indication). Finally, we calculated the time lapse in weeks from the date of conception (calculated using gestational age via the birth record) and the last HbA_1c_ test before pregnancy.

### Statistical Analysis

Statistical analysis was performed from August to November 2022. We first examined mean differences in preconception HbA_1c_ risk by gestational diabetes outcomes using 2-sample *t* tests and χ^2^ tests. Next, we used log binomial regression to estimate the unadjusted and adjusted relative risk (aRR) of gestational diabetes and adverse birth outcomes at first birth by preconception HbA_1c_ risk status. Adjusted models controlled for time lapse from the most recent preconception HbA_1c_ test, as well as maternal sociodemographic and prepregnancy characteristics.

Second, we used receiver operating characteristic (ROC) analysis to assess whether HbA_1c_ prediabetes thresholds extrapolated from adults were appropriate for use among adolescents for identifying the risk of gestational diabetes at first birth and to assess potential alternate cutoff points. The ROC curve plots the tradeoff between sensitivity (true positive rate) against its false positive rate (1 – specificity).^40^ The area under the curve (AUC) represents the average accuracy of measure in a single number ranging from 0 to 1, with an AUC of 0.5 indicative of a random guess and an AUC of 1 indicating perfect classification.^40^ We used ROC analysis to assess the sensitivity, specificity, and AUC of current thresholds (prediabetes indicated by HbA_1c_ ≥5.7% to <6.5%) used among adult populations.^1^ We next assessed alternate optimal HbA_1c_ cutoff point levels in projecting gestational diabetes among adolescents using the Youden index, which maximizes the tradeoff between sensitivity and specificity (sensitivity + specificity – 1) and the closest top left method (ie, the closest to [0,1] criteria on the ROC curve).^41,42^

Finally, we repeated ROC analyses across strata traditionally used to identify high-risk pregnancy for gestational diabetes: prepregnancy obesity status (obesity and overweight, normal weight, or underweight) and race and ethnicity. We performed data management using SAS Enterprise statistical software version 7.1.5 (SAS Institute) and statistical analysis using R statistical software version 1.6.9 (R Project for Statistical Computing). ROC analyses and estimation of the Youden index and ER method were performed using the pROC package in R statistical software version 1.18.0.^43^

Comparison of sociodemographic and clinical characteristics among those with and without preconception HbA_1c_ testing is shown in eTable 1 in Supplement 1. We conducted sensitivity analyses restricted to those aged 10 to 19 and 20 to 24 years in eTable 2, eTable 3, and eTable 4 in Supplement 1. Finally, although the previously discussed multivariable models adjusted for the time lapse since the most recent HbA_1c_ test before pregnancy, as an additional sensitivity analysis, we restricted the sample to those with an HbA_1c_ test within 12 months of pregnancy (8745 participants or 61.6% of the analytic sample) (eTable 3 in Supplement 1).

## Results

### Sociodemographic Characteristics by Preconception HbA_1c _Risk Status

In this sample of 14 302 adolescents and young adults (mean [SD] age, 22.10 [1.55] years) with at least 1 HbA_1c_ test before first birth, 883 (6.2%) were aged 10 to 19 years, and 13 419 (93.8%) were aged 20 to 24 years. Most in the sample were Hispanic (5869 individuals [41.0%]) followed by Black (4149 individuals [29.0%]), White (2583 individuals [18.1%]), Asian (1516 individuals [10.6%]), and other or unknown race and ethnicity (ie, Alaska Native, American Indian, Pacific Islander, multiple races, don’t know, or not reported; 185 individuals [1.3%]). More than one-third of the sample was born outside the US (5198 individuals [36.3%]), and the majority had Medicaid or no insurance (11 506 individuals [80.5%]). Most participants (10 612 individuals [74.2%]) had 1 preconception HbA_1c_ test only, 1273 (8.9%) had 2 tests, and 2417 (16.9%) had more than 2 tests. At last preconception test, the majority (11 407 individuals [79.7%]) had normoglycemia, and 2895 individuals (20.2%) had prediabetes. The mean (SD) time elapsed from last HbA_1c_ test and the start of pregnancy was 12.7 (12.1) months, with a median (IQR) of 9.0 (3.8-18.3) months (Table 1). There was no significant difference between whether the value of the last preconception test was considered normoglycemia or in the prediabetes level. The cumulative incidence of gestational diabetes was 6.6% (940 individuals), with higher mean HbA_1c_ values at last preconception test among those with incident gestational diabetes (5.7%; 95% CI, 5.7%-5.8%) than without (5.4%; 95% CI, 5.4%-5.4%). Those who identified as South and Southeast Asian were more likely to be classified as having prediabetes at the most recent preconception HbA_1c_ test (291 individuals [33.8%]), followed by those who identified as Black (1130 individuals [27.2%]), whereas White individuals had the lowest prediabetes prevalence (241 individuals [9.3%]). Those with prediabetes were more likely to have obesity (1014 individuals [29.4%]) than have normal weight (992 individuals [15.7%]). Those who used alcohol during pregnancy were also more likely to be classified with prediabetes before pregnancy (32 individuals [28.8%]) than those who did not report use (2858 individuals [20.2%]).

**Table 1.** Sociodemographic Characteristics of Birth Cohort by Preconception HbA_1c_ Risk Level^a^

Characteristic	Participants, No. (%)		
	Normoglycemia (n = 11 407)	Prediabetes (n = 2895)	Total (N = 14 302)
HbA_1c_ level, mean (SD), %	5.29 (0.25)	5.83 (0.49)	5.4 (3.2)
Age, mean (SD), y	22.10 (1.55)	22.10 (1.56)	22.10 (1.55)
Time lapse, mo^b^	12.7 (12.0)	12.6 (12.3)	12.7 (12.1)
Age group, y			
10-19	693 (78.5)	190 (21.5)	883 (6.2)
20-24	10 714 (79.8)	2705 (20.2)	13 419 (93.8)
Race and ethnicity			
Asian (all)	1084 (71.5)	432 (28.5)	1516 (10.6)
Asian (South/Southeast)	569 (66.2)	291 (33.8)	860 (6.01)
Black	3019 (72.8)	1130 (27.2)	4149 (29.0)
Hispanic	4823 (82.2)	1046 (17.8)	5869 (41.0)
White	2342 (90.7)	241 (9.3)	2583 (18.1)
Other or unknown^c^	139 (75.1)	46 (24.9)	185 (1.3)
Nativity			
US born	7345 (80.7)	1759 (19.3)	9104 (63.7)
Born outside the US	4062 (78.2)	1136 (21.9)	5198 (36.3)
Education level			
Less than high school	2013 (78.3)	559 (21.7)	2572 (18.0)
High school completion	3777 (80.1)	939 (19.9)	4716 (33.1)
Some college	3426 (78.5)	941 (21.6)	4367 (30.6)
College degree or higher	2156 (82.8)	448 (17.2)	2604 (18.3)
Insurance			
Medicaid or none	9125 (79.3)	2381 (20.7)	11 506 (80.5)
Private insurance	2186 (81.8)	486 (18.2)	2672 (18.7)
Prepregnancy body mass index^d^			
Underweight (<18.5)	725 (85.6)	122 (14.4)	847 (5.9)
Normal weight (18.5 to <25.0)	5337 (84.3)	992 (15.7)	6329 (44.3)
Overweight (25.0 to <30.0)	2854 (79.2)	750 (20.8)	3604 (25.2)
Obesity (≥30.0)	2440 (70.6)	1014 (29.4)	3454 (24.2)
Prepregnancy hypertension			
No	11 216 (80.0)	2805 (20.0)	14 021 (98.0)
Yes	191 (68.0)	90 (32.0)	281 (2.0)
Gestational diabetes at first live birth			
No	10 833 (81.1)	2529 (18.9)	13 362 (93.4)
Yes	574 (61.1)	366 (38.9)	940 (6.6)
Smoking (3 mo before pregnancy)			
No	11 100 (79.8)	2816 (20.2)	13 916 (97.3)
Yes	296 (78.9)	79 (21.1)	375 (2.6)
Alcohol use (during this pregnancy)			
No	11 287 (79.8)	2858 (20.2)	14 145 (98.9)
Yes	79 (71.2)	32 (28.8)	111 (0.80)

Abbreviation: HbA_1c_, hemoglobin A_1c_.

SI conversion factor: To convert HbA_1c_ to proportion of total hemoglobin, multiply by 0.01.

^a^ Sample consists of individuals who received an HbA_1c_ test before first pregnancy that resulted in live birth. Normoglycemia is defined as HbA_1c_ less than 5.7%. Prediabetes is defined as HbA_1c_ greater than or equal to 5.7% and less than 6.5%.

^b^ Pregnancy start date to last preconception test date.

^c^ Other or unknown categories includes Alaska Native, American Indian, Pacific Islander, multiple races, don’t know, or not reported.

^d^ Calculated as weight in kilograms divided by height in meters squared.

### aRR of Gestational Diabetes and Adverse Birth Outcomes by Preconception Prediabetes Status

Adjusting for sociodemographic and prepregnancy characteristics, those with preconception prediabetes according to original threshold values (HbA_1c_ ≥5.7% to <6.5%) had more than twice the likelihood of gestational diabetes (aRR, 2.21; 95% CI, 1.91-2.56) at first birth vs those with normoglycemia (HbA_1c_ <5.7%) (Table 2). Preconception prediabetes was also associated with slight but significant increases in the likelihood of a hypertensive disorder of pregnancy (aRR, 1.18; 95% CI, 1.03-1.35) and preterm delivery (aRR, 1.18; 95% CI, 1.02-1.37). The aRRs for cesarean delivery (aRR, 1.09; 95% CI, 0.99-1.20) and macrosomia (aRR, 1.13; 95% CI, 0.93-1.37) were also increased but not statistically significant. Main effect coefficients did not appreciably differ when the sample was restricted to those who had received an HbA_1c_ test in the 12 months preceding pregnancy (eTable 3 in Supplement 1). In sensitivity analyses restricted to those aged 10 to 19 years, there was no association of gestational diabetes with other adverse birth outcomes, with the exception of hypertensive disorders of pregnancy, which increased in magnitude (eTable 3 in Supplement 1). Among those aged 20 to 24 years, results were slightly greater for gestational diabetes and did not appreciably change for other outcomes.

**Table 2.** aRR of Gestational Diabetes and Adverse Birth Outcomes at First Birth Among Individuals by Preconception Diabetes Risk Threshold, Generalized Linear Models With Logit Link^a^

Outcome	aRR (95% CI)			
	HbA_1c_ 5.7% to <6.5%	HbA_1c_ 5.6% to <6.5%	HbA_1c_ 5.5% to <6.5%	HbA_1c_ 5.1% to <6.5%
Gestational diabetes first birth	2.21 (1.91-2.56)	2.14 (1.86-2.46)	1.95 (1.69-1.26)	1.30 (1.01-1.66)
Hypertensive disorders of pregnancy	1.18 (1.03-1.35)	1.23 (1.09-1.39)	1.18 (1.05-1.32)	1.13 (0.94-1.37)
Preterm delivery	1.18 (1.02-1.37)	1.16 (1.02-1.32)	1.13 (1.00-1.28)	0.86 (0.72-1.04)
Cesarean delivery	1.09 (0.99-1.20)	(1.04 (0.95-1.13)	1.07 (0.99-1.15)	1.06 (0.94-1.20)
Macrosomia	1.13 (0.93-1.37)	1.21 (1.02-1.43)	1.22 (1.04-1.43)	1.23 (0.94-1.59)

Abbreviations: aRR, adjusted relative risk; HbA_1c_, hemoglobin A_1c_.

SI conversion factor: To convert HbA_1c_ to proportion of total hemoglobin, multiply by 0.01.

^a^ Adjusted for age, race and ethnicity, nativity, education, insurance, prepregnancy body mass index category, chronic hypertension, smoking (3 months before pregnancy), alcohol use in pregnancy, and time lapse from last HbA_1c_ test.

### Optimal Preconception HbA_1c_ Thresholds for Gestational Diabetes Classification

The mean sensitivity and specificity of existing HbA_1c_ thresholds for classification of prediabetes (HbA_1c_ ≥5.7% to <6.5%) in estimating gestational diabetes at first birth were 38.9% (95% CI, 34.3%-40.3%) and 81.1% (95% CI, 80.4%-81.7%), respectively (Table 3). The optimal cutoff point in classifying gestational diabetes according to the Youden index was an HbA_1c_ level of 5.6% (sensitivity, 52.5%; specificity, 70.4%).^44,45^ An HbA_1c_ threshold further lowered to 5.5% would produce a sensitivity of 63.9% and specificity of 57.6%. Finally, lowering the threshold to an HbA_1c_ of 5.1% resulted in high sensitivity (92.2%) and very low specificity (12.5%).

**Table 3.** Cutoff Point Estimates for Preconception HbA_1c_ Levels in Estimation of Gestational Diabetes at First Birth Among Participants by Prepregnancy Body Mass Index Category

Example lower bound HbA_1c_ cutoff points (upper bound <6.5%)	Overall^a^		Obesity or overweight^b^		Normal weight^c^		Underweight^d^	
	Sensitivity, %	Specificity, %	Sensitivity, %	Specificity, %	Sensitivity, %	Specificity, %	Sensitivity, %	Specificity, %
5.1%	92.2	12.5	94.8	10.7	89.1	14.2	79.4	13.5
5.5%	63.9	57.6	71.1	52.1	57.4	62.4	32.4	61.7
5.6%^e^	47.9^e^	74.6^e^	59.3^e^	65.0^e^	47.9^e^	74.6^e^	20.6^e^	76.3^e^
5.7%	38.9	81.1	42.2	46.2	34.3	85.3	17.7	85.7
5.9%	20.1	94.1	26.2	91.5	19.0	95.6	5.8	96.3

Abbreviation: HbA_1c_, hemoglobin A_1c_.

SI conversion factor: To convert HbA_1c_ to proportion of total hemoglobin, multiply by 0.01.

^a^ Youden index, 1.23; closest top left, 0.336; and area under the curve, 0.648 (95% CI, 0.629-0.667).

^b^ Youden index, 1.24; closest top left, 0.288; and area under the curve, 0.664 (95% CI, 0.641-0.678).

^c^ Youden index, 1.23; closest top left, 0.336; and area under the curve, 0.634 (95% CI, 0.645-0.678).

^d^ Youden index, 1.05; closest top left, 0.783; and area under the curve, 0.490 (95% CI, 0.388-0.593).

^e^ Indicates optimal cutoff point, or the threshold that maximizes both sensitivity and specificity.

Using a wider HbA_1c_ threshold of 5.6% to less than 6.5% was associated with a similar increased risk of gestational diabetes (aRR, 2.21; 95% CI, 1.91-2.56) and similar risk of other adverse birth outcomes (Table 2). Further widening the HbA_1c_ threshold to lower cutoff points resulted in a significant but lower magnitude of risk for gestational diabetes. At the lowest HbA_1c_ cutoff point assessed (5.1%), preconception HbA_1c_ risk was associated with an increased risk of gestational diabetes (aRR, 1.30; 95% CI, 1.01-1.66) but no longer associated with the risk of preterm delivery (aRR, 0.86; 95% CI, 0.72-1.04). The risk of macrosomia did increase incrementally but was not statistically significant (aRR, 1.23; 95% CI, 0.94-1.59). In sensitivity analyses, we found that an HbA_1c_ threshold for identifying gestational diabetes at first birth was the same in the 10- to 19-year-old and 20- to 24-year-old age cohorts as the overall cohort (HbA_1c _5.6%); however, this resulted in a slightly higher sensitivity and specificity among the older age group (eTable 4 in Supplement 1).

### Optimal Preconception HbA_1c_ Thresholds by Prepregnancy Obesity Status and Race and Ethnicity

Table 3 and Table 4 report the sensitivity and specificity for varying HbA_1c_ thresholds by prepregnancy obesity status and race and ethnicity. We find that the optimal lower HbA_1c_ threshold (5.6%) did not vary depending on whether the adolescent had obesity and/or overweight, normal, or underweight weight, although sensitivity was slightly higher (59.3%) and specificity lower (65.0%) among those with obesity and/or overweight relative to normal weight (sensitivity, 47.9%; specificity, 74.6%). Among those underweight, sensitivity (20.6%) and specificity (76.3%) were notably lower at the same threshold. We did, however, find differences in optimal cutoff point by race and ethnicity, with a lower HbA_1c_ threshold (5.5%) identified among Hispanic persons (sensitivity, 60.4%; specificity, 58.5%). For all other racial and ethnic groups examined, the optimal cutoff point was the same (HbA_1c_ 5.6%). At this cutoff point, there was an observed higher sensitivity and lower specificity among Black (sensitivity, 62.0%; specificity, 62.1%) and South and Southeast Asian persons (sensitivity, 66.2%; specificity, 57.4%) than White persons (sensitivity, 42.4%; specificity, 84.0%).

**Table 4.** Cutoff Point Estimates for Preconception HbA_1c_ Levels in Estimation of Gestational Diabetes at First Birth Among Participants by Race and Ethnicity

Example lower bound HbA_1c_ cutoff points (upper bound <6.5%)	Black^a^		Hispanic^b^		South and Southeast Asian^c^		White^d^	
	Sensitivity, %	Specificity, %	Sensitivity, %	Specificity, %	Sensitivity, %	Specificity, %	Sensitivity, %	Specificity, %
5.1%	93.0	11.3	90.4	12.8	96.9	4.5	89.0	17.3
5.5%	72.5	49.9	60.4	58.5	75.4	40.1	51.7	73.3
5.6%	62.0	62.1	47.2	71.5	66.2	57.4	42.4	84.0
5.7%	46.7	73.8	31.5	83.0	45.9	75.2	26.8	35.0
5.9%	31.9	89.9	19.5	94.6	29.2	90.5	13.6	97.9

Abbreviation: HbA_1c_, hemoglobin A_1c_.

SI conversion factor: To convert HbA_1c_ to proportion of total hemoglobin, multiply by 0.01.

^a^ Youden index, 1.20; closest top left, 0.288; and area under the curve, 0.664 (95% CI, 0.625-0.703).

^b^ Youden index, 1.19; closest top left, 0.329; and area under the curve, 0.626 (95% CI, 0.593-0.659).

^c^ Youden index, 1.24; closest top left, 0.378; and area under the curve, 0.648 (95% CI, 0.602-0.695).

^d^ Youden index, 1.26; closest top left, 0.358; and area under the curve, 0.664 (95% CI, 0.608-0.720).

## Discussion

In this adolescent and young adult preconception HbA_1c_ birth cohort, we found that preconception prediabetes (HbA_1c_ ≥5.7% to <6.5%) was associated with more than twice the likelihood of gestational diabetes at first birth as well as higher risk of hypertensive disorders of pregnancy and preterm birth. We also documented that a lower threshold for prediabetes (HbA_1c_ 5.6%) than the current adult threshold (HbA_1c_ 5.7%) was more ideal to identify gestational diabetes at first birth among adolescents and young adults. Although the empirical threshold did not vary by prepregnancy obesity status, a slightly lower cutoff (HbA_1c _5.5%) was found to be optimal among Hispanic individuals.

This study adds to the scant evidence base on the role of preconception HbA_1c_ levels among adolescents and young adults on cardiometabolic dysfunction in pregnancy. Currently, the American Diabetes Association (ADA) offers preconception cardiometabolic risk counseling tailored for adolescents with pregestational diabetes.^46^ However, those with subclinical diabetes risk and no history of diabetes are unlikely to be engaged in routine diabetes care and to receive preconception counseling on glycemic control independent of BMI. Given that most pregnancies among adolescents and young adults are unwanted or mistimed, the reach of preconception counseling efforts is limited.^47^ Prior research has found that both pharmacological and lifestyle interventions in pregnancy may be too little, too late to substantially improve maternal cardiometabolic health.^48^ Given the demonstrated risk subclinical HbA_1c_ levels have for the development of gestational diabetes and, in turn, life course cardiometabolic risk, our results suggest that efforts to incorporate cardiometabolic risk counseling into routine health services, such as annual school checkups, are warranted. Prior evidence that adolescent childbearing is more likely in geographic areas marked by high socioeconomic disadvantage and a shortage of health professionals^49^ further underscores the potential of efforts to expand more generalized HbA_1c_ screening into routine preventive services.

Although our results highlight the clinical risks associated with subclinical preconception HbA_1c_ levels among adolescents and young adults, we also found that extrapolating HbA_1c_ prediabetes thresholds from adults was not the most appropriate cutoff point for identifying the association with a future gestational diabetes pregnancy. Even when adopting a lower prediabetes cutoff for HbA_1c_ of 5.6% vs 5.7%, approximately one-half of gestational diabetes cases would be missed. However, a lower HbA_1c_ threshold considered at risk would still identify a greater proportion of future gestational diabetes cases, at the cost of intervening among individuals who may not develop the condition. Prior literature^50,51^ has documented low awareness among young people, including those with diabetes, on the associated risks between obesity, poor cardiometabolic health, and pregnancy health. Taken together with results from this study, bringing attention to the role of heart health in pregnancy among young people is likely to have fewer drawbacks than lack of action.^52^

Notably, as of 2018, the ADA has broadened diabetes screening among asymptomatic children and adolescents to include those who are overweight with only 1 additional risk factor.^21,53^ Our results support these more generalized screening guidelines. We found similar HbA_1c_ risk thresholds for estimating gestational diabetes among Black, South and Southeast Asian, and White individuals (HbA_1c_ 5.6%), with a slightly broader threshold among Hispanic persons (HbA_1c_ 5.5%). We also identified the same HbA_1c_ thresholds across BMI subgroups for identifying gestational diabetes risk and by age group (10-19 years vs 20-24 years). Previous guidelines^1^ have included the age period 20 to 24 years in adult recommendations, whereas our findings suggest a slightly lower threshold (HbA_1c_ 5.6%) than that of the ADA. Taken together, lower HbA_1c_ prediabetes thresholds than those applied to adults were optimal for identifying the likelihood of a future gestational diabetes pregnancy across traditional diabetes risk groups. These results support the distinction between weight and level of glycemia in adolescent cardiometabolic risk and underscore the need for adolescent and young adult health guidelines to emphasize health behaviors (ie, routine physical activity) to optimize blood glucose levels before pregnancy outside of weight loss alone.^47^

### Strengths and Limitations

Strengths of this study include use of the APPLE Cohort, a large, multiethnic cohort of primiparous adolescents and young adults who were screened for HbA_1c_ before conception and had no history of diabetes. We combined information from the birth registry and hospital diagnoses codes to enhance the sensitivity of measures of maternal and neonatal clinical comorbidity where possible. However, several limitations exist. All covariates are subject to misclassification. However, previous validation of birth registry data supports the accuracy of key variables such as gestational age, which was used to define the preconception period.^54^ Information on lifestyle or interventions received, which may influence glycemia, were not available in this dataset, nor were reasons for undergoing HbA_1c_ screening. Furthermore, the time between the last HbA_1c_ test received before pregnancy and the start of pregnancy was not uniform. We performed a sensitivity analysis to restrict the sample to those who were tested in the last 12 months in eTable 2 in Supplement 1 and found results did not appreciably change.

Finally, results are representative of adolescents and young adults who were HbA_1c_ tested before pregnancy. Previous analyses of APPLE cohort data found higher representation of women with gestational diabetes in the HbA_1c_ Registry.^14^ Our sample is therefore more likely to be representative of an adolescent population who met established risk criteria and may be at higher risk of gestational diabetes (eTable 1 in Supplement 1).^21^ For example, we document slightly larger magnitude of association between preconception glucose levels and the likelihood of gestational diabetes than the Young Finns study.^55^ In that study, primiparous women with increased glucose levels approximately 10 weeks before the first prenatal visit had an increased aRR for gestational diabetes that was not statistically significant (aRR, 1.38; 95% CI, 0.92-2.06).^55^ Extensive bias analysis to adjust for differential screening practices in the APPLE Cohort, however, found that adjusting for screening bias only slightly attenuated study findings.^14^ We also did not have information on all clinically relevant measures such as whether preterm birth was spontaneous or any pharmacological or lifestyle interventions. Although continued work is needed in additional adolescent cohorts, results nevertheless suggest that the lack of uniform preconception prediabetes treatment guidelines for pregnant adolescents may represent a missed opportunity to intervene on excess cardiometabolic risk before conception and potential pregnancy-related complications with implications for life course health.

## Conclusions

This study adds to the scant literature on the validity of using subclinical HbA_1c_ levels in adolescence and young adulthood to detect risk for cardiometabolic complications in pregnancy. We found adolescent and young adult preconception HbA_1c_ levels within a prediabetes risk threshold of HbA_1c_ greater than or equal to 5.6% to less than 6.5% is a potentially modifiable risk factor that can be targeted for reducing the likelihood of gestational diabetes and adverse maternal and neonatal outcomes at first birth. Given alarming trends in adolescent obesity and diabetes risk and the high prevalence of unplanned pregnancies in adolescence and young adulthood, our results support expanded preconception screening as a mechanism to intervene on excess cardiometabolic risk earlier in the life course.

## Notes

Supplement 1. **eTable 1. **Comparison of Study Sample With and Without a Preconception A1c Test at First Live Birth, Among Those With no History of Diabetes **eTable 2. **Sample Sociodemographic Characteristics and Adverse Birth Outcomes Among Those With at Least 1 Preconception HbA1c Test Before Pregnancy by Age Group **eTable 3. **Adjusted Relative Risk of Gestational Diabetes and Adverse Birth Outcomes by Preconception Prediabetes Status by Timing of Testing and Age Group: Sensitivity Analysis **eTable 4. **Cut Point Estimates and AUC Values for Preconception Hemoglobin HbA1c Levels in Estimation of Gestational Diabetes at First Birth Stratified by Age Group

Supplement 2.
Data Sharing Statement
