# Supplementary material for: Preconception HbA1c Levels in Adolescents and Young Adults and Adverse Birth Outcomes
Source: JAMA Netw Open. 2024 Sep 24;7(9):e2435136. doi: 10.1001/jamanetworkopen.2024.35136 (PMC11423169; doi:10.1001/jamanetworkopen.2024.35136)
Supplement: Supplement 1. — eTable 1. Comparison of Study Sample With and Without a Preconception A1c Test at First Live Birth, Among Those With no History of Diabetes eTable 2. Sample Sociodemographic Characteristics and Adverse Birth Outcomes Among Those With at Least 1 Preconception HbA1c Test Before Pregnancy by Age Group eTable 3. Adjusted Relative Risk of Gestational Diabetes and Adverse Birth Outcomes by Preconception Prediabetes Status by Timing of Testing and Age Group: Sensitivity Analysis eTable 4. Cut Point Estimates and AUC Values for Preconception Hemoglobin HbA1c Levels in Estimation of Gestational Diabetes at First Birth Stratified by Age Group [file jamanetwopen-e2435136-s001.pdf]

Supplemental Online Content

McCarthy KJ, Liu SH, Kennedy J, et al. Preconception HbA_{1c} levels in adolescents and young adults and adverse birth outcomes. *JAMA Netw Open*. 2024;7(9):e2435136. doi:10.1001/jamanetworkopen.2024.35136

eTable 1. Comparison of Study Sample With and Without a Preconception A1c Test at First Live Birth, Among Those With no History of Diabetes

eTable 2. Sample Sociodemographic Characteristics and Adverse Birth Outcomes Among Those With at Least 1 Preconception HbA1c Test Before Pregnancy by Age Group

eTable 3. Adjusted Relative Risk of Gestational Diabetes and Adverse Birth Outcomes by Preconception Prediabetes Status by Timing of Testing and Age Group: Sensitivity Analysis

eTable 4. Cut Point Estimates and AUC Values for Preconception Hemoglobin HbA1c Levels in Estimation of Gestational Diabetes at First Birth Stratified by Age Group

This supplemental material has been provided by the authors to give readers additional information about their work.

© 2024 McCarthy KJ et al. *JAMA Network Open.*

eTable 1. Comparison of Study Sample With and Without a Preconception A1c Test at First Live Birth, Among Those With no History of Diabetes

	No pre-conception A1c test (N=148,767)	1+ Preconception A1c test (N=14,302)
Mean A1c (SD) (N=73,431)	5.3 (0.4)	5.4 (0.3)
Mean age (SD)	20.8 (2.3)	22.1 (1.6)
**Age group**	*N (Col %)*	*N (Col %)*
10 to 19 years	40584 (27.3)	883 (6.2)
20 to 24 years	108068 (72.7)	13419 (93.8)
**Race/ethnicity**		
Asian (All)	14566 (9.8)	1516 (10.6)
Black	39701 (26.7)	4149 (29.0)
Hispanic	65669 (44.1)	5869 (41.0)
South/SE Asian	4632 (3.1)	860 (6.01)
Other/Unknown [a]	2159 (1.5)	185 (1.3)
White	26672 (17.9)	2583 (18.1)
**Nativity**		
US born	88351 (59.4)	9104 (63.7)
Foreign born	60416 (40.6)	5198 (36.3)
**Education level**		
Less than high school	48275 (32.6)	2572 (18.0)
High school completion	48379 (32.7)	4716 (33.1)
Some college	34330 (23.2)	4367 (30.6)
College degree or higher	17173 (11.6)	2604 (18.3)
**Insurance**		
Medicaid or none	125708 (85.5)	11506 (80.5)
Private insurance	21369 (14.5)	2672 (18.7)
**Pre-pregnancy BMI[a]**		
Underweight (< 18.5)	11813 (8.0)	847 (5.9)
Normal weight (18.5 - < 25.0)	81193 (55.2)	6329 (44.3)
Overweight (25.0 - < 30.0)	33052 (22.5)	3604 (25.2)
Obesity (≥ 30.0)	21130 (14.4)	3454 (24.2)
**Pre-pregnancy hypertension**		
No	146934 (98.8)	14021 (98.0)
Yes	1833 (1.2)	281 (2.0)
**GDM[a] at first live birth**		
No	142584 (95.8)	13362 (93.4)
Yes	6183 (4.2)	940 (6.6)
**Smoking (3 months prior to pregnancy)**		
No	147723 (99.3)	13916 (97.3)
Yes	962 (0.7)	375 (2.6)
**Alcohol use (during this pregnancy)**		
No	145061 (97.5)	14145 (98.9)
Yes	3697 (2.5)	111 (0.80)

[a] Other/Unknown racial/ethnic categories include: Alaska Native, Native American, Pacific Islander, Multiple races, 'Don't Know' or not reported categories; BMI=body mass index (kg/m^2); GDM=gestational diabetes.

© 2024 McCarthy KJ et al. *JAMA Network Open.*

eTable 2. Sample Sociodemographic Characteristics and Adverse Birth Outcomes Among Those With at Least 1 Preconception HbA_{1c} Test Before Pregnancy by Age Group

	Ages 10-19 Years		Ages 20-24 Years		Total
	Normo-glycemia (A1c<5.7) (N= 693)	Prediabetes (5.7≤A1c< 6.5) (N=190)	Normo-glycemia (A1c<5.7) (N= 10,714)	Prediabetes (5.7≤A1c< 6.5) (N=2,704)	(N=14,302)
	Row (%)	*Row (%)*	*Row (%)*	*Row (%)*	*Column (%)*
Mean A1c (SD)	5.3 (0.2)	5.8 (0.2)	5.3 (0.3)	5.8 (0.2)	5.4 (3.2)
Mean age (SD)	18.8 (0.4)	18.8 (0.5)	22.3 (1.3)	22.4 (1.3)	22.1 (1.6)
**Race/ethnicity**					
Asian (All)	23 (71.9)	9 (28.1)	1061 (71.5)	423 (28.5)	1516 (10.6)
South/SEAsian					860 (6.01)
Black	207 (75.0)	69 (25.0)	2812 (72.6)	1061 (27.4)	4149 (29.0)
Hispanic	367 (78.3)	102 (21.8)	4456 (82.5)	944 (17.5)	5869 (41.0)
Other/Unknown[a]	14 (82.4)	3 (17.7)	125 (74.4)	43 (25.6)	185 (1.3)
White	82 (92.1)	7.9 (9.0)	2260 (90.6)	234 (9.4)	2583 (18.1)
**Nativity**					
US born	504 (79.3)	132 (20.8)	6841 (80.8)	1627 (19.2)	9104 (63.7)
Foreign born	189 (76.5)	58 (23.5)	3873 (78.2)	1078 (21.8)	5198 (36.3)
**Education level**					
Less than high school	286 (78.6)	78 (21.4)	1727 (78.2)	481 (21.8)	2572 (18.0)
High school completion	260 (78.3)	72 (21.7)	3517 (80.2)	867 (19.8)	4716 (33.1)
Some college	132 (77.2)	39 (22.8)	3294 (78.5)	902 (21.5)	4367 (30.6)
College degree or higher	14 (93.3)	1 (6.7)	2142 (82.7)	447 (17.3)	2604 (18.3)
**Insurance**					
Medicaid or none	611 (78.3)	169 (21.7)	8514 (79.4)	2212 (20.6)	11506 (80.5)
Private insurance	75 (79.0)	20 (21.1)	2111 (81.9)	466 (18.1)	2672 (18.7)
**Pre-pregnancy BMI[a]**					
Underweight (<18.5)	42 (82.4)	9 (17.7)	683 (85.8)	113 (14.2)	847 (5.9)
Normal weight (18.5< 25.0)	313 (81.3)	72 (18.7)	5024 (84.5)	920 (15.5)	6329 (44.3)
Overweight (25.0< 30.0)	191 (78.9)	51 (21.1)	2663 (79.2)	699 (20.8)	3604 (25.2)
Obesity (≥30.0)	142 (71.0)	58 (29.0)	2298 (70.6)	956 (29.4)	3454 (24.2)
**Pre-pregnancy hypertension**					
No	687 (78.8)	185 (21.2)	10529 (80.1)	2620 (19.9)	14021 (98.0)
Yes	6 (54.5)	5 (45.5)	185 (68.5)	85 (31.5)	281 (2.0)
**GDM[a] at first live birth**					
No	662 (78.7)	179 (21.9)	10171 (81.2)	2350 (18.8)	13362 (93.4)
Yes	31 (73.8)	11 (26.2)	543 (60.5)	355 (39.5)	940 (6.6)
**Hypertensive disorder of pregnancy**					
No	637 (79.6))	163 (20.4)	9766 (80.4)	2388 (19.7)	12954 (90.6)
Yes	56 (67.5)	27 (32.5)	948 (74.9)	317 (25.1)	1348 (9.4)
**Preterm delivery**					
No	625 (78.3)	173 (21.7)	9837 (80.3)	2418 (19.7)	13053 (91.3)
Yes	68 (80.0)	17 (20.0	877 (75.3)	287 (24.7)	1249 (8.7)
**Cesarean section**					
No	517 (78.0)	146 (22.0)	7867 (81.1)	1830 (18.9)	10360 (72.4)
Yes	176 (80.0)	44 (20.0)	2847 (76.5)	875 (23.5)	3942 (27.6)
**Macrosomia**					
No	659 (78.5)	181 (21.6)	10232 (80.0)	2560 (20.0)	13632 (95.3)
Yes	34 (79.1)	9 (20.9)	482 (76.9)	145 (23.1)	670 (4.7)

[a] Other/Unknown racial/ethnic categories include: Alaska Native, Native American, Pacific Islander, Multiple races, 'Don't Know' or not reported categories; BMI=body mass index (kg/m^2); GDM=gestational diabetes.

© 2024 McCarthy KJ et al. *JAMA Network Open.*

eTable 3. Adjusted[a] Relative Risk of Gestational Diabetes (GDM) and Adverse Birth Outcomes by Preconception Prediabetes Status by Timing of Testing and Age Group: Sensitivity Analysis

	**Ages 10–24 tested in 12 months preceding pregnancy (n=8,745)**	**Ages 10- to 19 at last preconception A1c test (n=833)**	**Ages 20- to 24 at last preconception A1c test (n=13,419)**
	**aRR (95% CI)**	**aRR (95%CI)**	**aRR (95%CI)**
GDM first birth	2.28 (1.90, 2.74)	1.09 (0.53, 2.26)	2.45 (2.12, 2.83)
Hypertensive disorders of pregnancy	1.15 (0.96, 1.38)	1.71 (1.03, 2.85)	1.16 (1.01, 1.34)
Preterm delivery	1.22 (1.01, 1.48)	0.91 (0.50, 1.64)	1.13 (1.11, 1.50)
Cesarean section	1.09 (0.96, 1.23)	0.83 (0.56, 1.23)	1.18 (1.07, 1.30)
Macrosomia	1.06 (0.83, 1.36)	0.91 (0.42, 1.97)	1.07 (0.88, 1.31)

[a]Adjusting for age, race/ethnicity, nativity, education, insurance, pre-pregnancy body mass index (BMI) (kg/m^2), chronic hypertension, smoking (3months prior to pregnancy), alcohol use in pregnancy, time lapse from last a1c test.

© 2024 McCarthy KJ et al. *JAMA Network Open.*

eTable 4. Cut Point Estimates and AUC Values for Preconception Hemoglobin HbA_{1c} Levels in Estimation of Gestational Diabetes at First Birth Stratified by Age Group

	**Ages 10-24 Years**			**Ages 10-19 Years**			**Ages 20-24 Years**		
	Point	Se	Sp	Point	Se	Sp	Point	Se	Sp
Example lower bound A1c cut-off points (upper bound<6.5) with Se and Sp values	5.1	92.2	12.5	5.1	90.5	12.3	5.1	88.2	12.5
	5.5	63.9	57.6	5.5	45.2	60.0	5.5	64.8	57.4
	**5.6**	**47.9**	**74.6**	**5.6**	**35.7**	**66.7**	**5.6**	**53.2**	**70.7**
	5.7	38.9	81.1	5.7	26.2	78.7	5.7	39.5	81.2
	5.9	20.1	94.1	5.9	14.3	93.0	5.9	20.4	94.2
Youden index[a]	0.23			0.02			0.24		
Closest top left[a]	0.336			0.305			0.519		
AUC[a] (95% CI)	0.648 (0.629, 0.667)			0.512 (0.437, 0.587)			0.604 (0.588 0.620)		

[a]Values correspond with optimal cutpoint which is indicated in bolded text, i.e., threshold which maximizes both sensitivity and specificity. Bolded text indicates 'optimal cutpoint', Point = cut-point, Se = sensitivity, Sp= specificity, AUC = area under the curve.

© 2024 McCarthy KJ et al. *JAMA Network Open.*
