# Supplementary material for: Preconception HbA1c Levels in Adolescents and Young Adults and Adverse Birth Outcomes
Source: JAMA Netw Open. 2024 Sep 24;7(9):e2435136. doi: 10.1001/jamanetworkopen.2024.35136 (PMC11423169; doi:10.1001/jamanetworkopen.2024.35136)
Supplement: Supplement 2. — Data Sharing Statement [file jamanetwopen-e2435136-s002.pdf]

Data Sharing Statement

McCarthy. Preconception HbA_{1c} Levels in Adolescents and Young Adults and Adverse Birth Outcomes. *JAMA Netw Open*. Published September 24, 2024.
doi:10.1001/jamanetworkopen.2024.35136

Data
Data available: No

Additional Information
Explanation for why data not available: Data are restricted access via the NYC Department of Health and Mental Hygiene and will be made available upon reasonable request via approval.
